# Bone fragility and sarcoidosis: An underestimated relationship

**DOI:** 10.3389/fmed.2022.1026028

**Published:** 2022-11-17

**Authors:** Carla Caffarelli, Paolo Cameli, Antonella Al Refaie, Elisa Giglio, Giulio Manzana, Caterina Mondillo, Yari Noacco, Carmela Olivieri, Elena Bargagli, Stefano Gonnelli

**Affiliations:** ^1^Section of Internal Medicine, Department of Medicine, Surgery and Neuroscience, University of Siena, Siena, Italy; ^2^Respiratory Diseases and Lung Transplantation Unit, Department of Medicine, Surgery and Neuroscience, University of Siena, Siena, Italy

**Keywords:** sarcoidosis, osteoporosis, bone mineral density, vertebral fractures, DLCO

## Abstract

**Introduction:**

Sarcoidosis is a chronic multisystem inflammatory disease which may affect any organ. Also bone can be involved both directly and indirectly. Data on BMD values and fragility fractures in sarcoidosis patients are few and heterogeneous. This study aimed to characterized the presence of fracture and the relative risk factors in patients with sarcoidosis.

**Materials and methods:**

In this single center cross-sectional study we evaluated 252 sarcoidosis patients (54.7 ± 12.1 years) compared to sex-and age matched healthy controls. We measured BMD at lumbar spine, at femoral neck and at total hip. Moreover, the presence of fragility fractures was collected during osteoporosis visit and all radiological images were examined for the presence of any vertebral fracture according to Genant’s method’s. Lung function measurements, including forced expiratory volume in one second (FEV1), forced vital capacity (FVC), FEV1/FVC, and diffusion capacity for carbon monoxide (DLCO) were assessed.

**Results:**

Bone Mineral Density T-scores were lower in patients affected by sarcoidosis with respect to those obtained in healthy controls, but the difference was statistically significant only for BMD-LS (*p* < 0.01) and BMD-TH (*p* < 0.05). Moreover, BMD values at all skeletal sites were significantly associated with DLCO (%) (*p* < 0.05). The prevalence of fragility fracture was higher in patients with sarcoidosis than in healthy controls (30.6 vs. 12.3%). The patients with ≥3 vertebral fracture had lower values of FVC (%), FEV1 (%), and DLCO (%). Multiple regression analyses showed that BMI was positively associated with fragility fracture, while BMD-TH, DLCO(%) and therapy use was negatively associated.

**Conclusions:**

Vertebral fractures represent a frequent complication in patients with sarcoidosis. Furthermore, the number of vertebral fractures was linked with a worsening in pulmonary functional tests. Therefore, the degree of severity of the sarcoidosis disease appears to be the main determinant of bone fragility.

## Introduction

Sarcoidosis is a chronic multisystem inflammatory disease characterized by the accumulation of CD4 + T helper lymphocytes and active macrophages that promote the formation of non-caseous granulomas in one or more organs or tissues. The etiology of sarcoidosis is still unknown. The most accredited hypothesis is that sarcoidosis is a multifactorial pathology in which subjects with a certain genetic susceptibility trigger and altered immune response after exposure to environmental or even self-type antigens. Macrophages, T lymphocytes and cytokines (IL-1, IL-6, INF-γ, TNF-α) play an important role in the pathogenesis of sarcoidosis (1).

Sarcoidosis can occur at any age, more frequently in adults between 30 and 50 years, and presents a higher incidence in women and in Scandinavian populations (1, 2).

However, the data on the epidemiology of sarcoidosis are still partial; the prevalence of the disease is 4.7–64.0 in 100,000, and annual incidence is 1.0–35.5 in 100,000 (2). In Italy, recent epidemiological data indicate an incidence of 49 cases per 100,000 inhabitants (3). Clinical features of sarcoidosis are extremely heterogeneous and this often makes difficult the diagnosis. General symptoms such as dyspnea, fatigue, fever and sweating are frequently observed. Sarcoidosis can affect any organ, the most frequently affected organs are: lung (90%), lymph nodes (10–20%), skin (15%), eye (10–30%), musculoskeletal system (25–30%), and liver (10–30%) (4, 5). The clinical course of sarcoidosis is also very variable; in fact in most cases there is spontaneous remission while in others, important complications are observed. The mortality rate is about 7% within a follow-up period of 5 years. More than 10% of patients with pulmonary sarcoidosis develop progressive disease, and over 60% of deaths are due to advanced lung involvement (6, 7).

In sarcoidosis bone tissue can be involved both directly and indirectly. Direct bone involvement is rare (3–12%) and often occurs in patients with advanced disease and multi-organ involvement (8). Moreover, bone health can be indirectly compromised through several mechanisms, not fully clarified, that link bone and sarcoidosis. In fact, alterations in calcium metabolism are common in patients with sarcoidosis and hypercalcemia and hypercalciuria may be part of bone involvement. Depending on the studies and population studied, hypercalcemia affects from 3 up to 18% of patients with sarcoidosis whereas hypercalciuria may affect 20–40% of patients (9–11). Other studies have shown an increase in both bone formation and bone resorption markers in patients suffering from sarcoidosis so suggesting an acceleration of bone turnover (12).

However, despite the alterations in bone metabolism and the use of glucocorticoids as first-line drugs, patients with sarcoidosis do not have an evident reduction in bone mineral densitometry (BMD) compared to normal subjects. Indeed, data on BMD values in sarcoidosis patients are few and heterogeneous. Some previous studies found moderately reduced BMD values in sarcoidosis patients both treated and untreated with glucocorticoids (13–15). On the contrary, some more recent studies (cross-sectional and longitudinal) have not found bone loss even in patients on glucocorticoid therapy (16–18).

Despite this, several previous studies have demonstrated a high prevalence of fragility fractures in patients with sarcoidosis (16, 17). In particular, a recent study conducted on a large cohort of patients participating to the Rochester Epidemiology Project, reported that patients with sarcoidosis have a higher incidence of fragility fractures than a control population (19). The study by Bours et al., reported that sarcoidosis patients who had received prednisone (>10 mg/day) presented a doubled risk of any fragility fractures (20). On the contrary, a case-control study carried out using the Danish Hospital Discharge Registry reported that sarcoidosis patients not treated with steroids did not have an increased risk of fragility fractures (21).

This single-center, cross-sectional study aimed to:

1. to evaluate the presence of vertebral and non-vertebral fragility fractures in a large cohort of sarcoidosis patients compared with to sex- and age-matched healthy controls,
2. to identify the risk factors associated with the presence of clinical and morphological vertebral fractures.

## Materials and methods

### Study population

We selected 252 patients (age range 31–83 years; mean age 54.7 ± 12.0 years) affected by sarcoidosis, referred to the Regional Referral Center for Sarcoidosis and other Interstitial Lung Diseases at Siena University Hospital (Siena, Italy) from January 2018 to December 2021. Diagnosis of sarcoidosis was performed through a multidisciplinary evaluation according to the diagnostic criteria of the ATS/ERS/JRS/ALAT guidelines (6, 22). All these patients underwent an evaluation of bone status at the outpatient Clinic for Osteoporosis of the Department of Internal Medicine at the University Hospital (Siena, Italy). Patients with sarcoidosis suffering from secondary forms of osteoporosis such as chronic renal failure (with GFR less than 30 mL/min), hyperparathyroidism, hypothyroidism, and known malignancy were excluded.

Sarcoidosis patients with a history of alcoholism or who had been taking for long periods or were taking drugs that interfere with bone metabolism such as anabolic steroids, gonadic hormones, anticonvulsants, vitamin D analogs, teriparatide, parathyroid hormone, denosumab, or bisphosphonates were excluded. All patients underwent a detailed medical and drug history regarding to smoking habit, comorbidities and any drugs taken for the treatment of sarcoidosis. At the time of data collection, 160 patients (63%) were on pharmacological therapies including prednisone (*n* = 136; 85%) and disease-modifying anti-rheumatic drugs (DMARDs, such as methotrexate, azathioprine, hydroxychloroquine) (*n* = 72, 45%).

Moreover, height and weight were measured in a standardized manner and subsequently the BMI was calculated as weight in kilograms divided by the square of height in meters.

Age and sex-matched healthy controls were recruited from a sub-group of individuals living in the area of Siena (Italy), who had been participating in a larger epidemiological study (23). Written consent was obtained from all participants, and the study was approved by the Ethics Committee of Siena University Hospital.

### Biochemical parameters

In all subjects, the serum levels of calcium (Ca), phosphate (P), creatinine (Cr), alkaline phosphatase (ALP), intact parathyroid hormone (PTH), and 25-Hydroxyvitamin D (25OHD) were measured in the morning under fasting conditions. Urinary calcium, phosphate and creatinine were determined by a colorimetric method (Cobas C311 analyzer, Roche Diagnostics, USA) in 24-h urine samples. Serum 25OHD was determined by a radioimmunometric method (25-Hydroxyvitamin D, DiaSorin, MN, USA). In our Institution the intra- and inter-assay coefficients of variation for 25OHD were 6.8 and 9.2%, respectively. Serum PTH was assessed by an immunoradiometric assay (DiaSorin, Saluggia, Italy). The results were expressed in picograms per milliliter, and the intra and inter-assay coefficients of variation were 3.6 and 4.9%, respectively.

### Densitometric measurements

Across the study population we evaluated BMD at the lumbar spine [LS-BMD] and at femoral subregions (femoral neck [FN-BM] and total hip [TH-BM]) using a dual-energy X-ray absorptiometry device (Lunar Prodigy; GE Healthcare, Waukesah, WI, USA). Diagnosis of osteoporosis and osteopenia was made in accordance with the World Health Organization (WHO) definition. In particular, a T-score value lower than −2.5 identifies a condition of osteoporosis, while T-score values between −1 and −2.5 identifies a condition of osteopenia, T-score values higher than −1 identify a condition of normality. For the calculation of all T-scores sex-matched Italian reference data were used.

### Vertebral fractures assessment

The information on history of previous fracture was collected during osteoporosis visit. In particular, details of fracture site, including spine, hip, wrist, clavicle, upper arm/shoulder, rib, pelvis, ankle, upper leg, and lower leg were assessed. In addition to the x-ray examinations that the patients showed during the osteoporotic visit, we checked all the reports in the hospital Carestream database for the diagnosis of vertebral fractures. Specially, chest x-ray in latero-lateral projection, Magnetic Resonance Imaging (MRI), chest high resolution computed tomography (HRCT), Computed Tomography (CT), and Computed Tomography with 18F-fluorodeoxyglucose (FDG PET-CT) reports were also reviewed. Namely, all radiological images were examined for the presence of any vertebral fracture according to Genant’s method’s (24). The morphometry was conducted by examining the section of the spine from the fourth thoracic vertebra (T4) to the fourth lumbar vertebrae (L4); in each vertebral body we marked six points, corresponding to the four corners and the midpoints of the endplates. The anterior (Ha), mid-vertebral (Hm), and posterior (Hp) heights of each vertebra were measured and the three ratios, Ha/Hp, Hm/Hp, and Hp/Hp-below, were calculated (24). Two Authors (GS and CC) independently evaluated the presence of vertebral fractures. In cases of divergent opinions, consensus was reached by discussion with a radiologist.

### Lung function assessment

All the participants underwent pulmonary function tests performed according to the American Thoracic Society/European Respiratory Society (ATS/ERS) standards (25, 26), using a Jaeger body plethysmograph with corrections for temperature and barometric pressure. Forced vital capacity (FVC), forced expiratory volume in 1 s (FEV1), FEV1/FVC and lung diffusion capacity for carbon monoxide (DLCO) were assessed. All subjects with sarcoidosis performed a chest X-ray with radiological staging according to Scadding criteria (27). The radiological classification was linked to sample detection in a standard manner according to widely accepted criteria: stage 0, normal; stage 1, bilateral hilar adenopathy without parenchymal involvement; stage 2, bilateral adenopathy and parenchymal infiltration; stage 3, parenchymal infiltration; and stage 4, pulmonary fibrosis associated with sarcoidosis.

### Statistical analysis

The normality of the distribution of the outcome variables was assessed by the Kolmogorov–Smirnov test. All the variables were normally distributed and were expressed as mean ± SD. The significance between the means was tested using Student’s *t*-test. Categorical variables were compared by Chi-square test or Fisher’s exact test, as appropriate. The correlations between the groups were analyzed with the Pearson’s correlation test. Analysis of variance (ANOVA) was used to analyze the association of lung functionally test and the number of vertebral fragility fractures.

Multiple linear regression models were used to assess the association of independent predictors such as sex, age, BMI, creatinine, vitamin D, BMD-LS, BMD-FN, BMD-TH, FVC (%), FEV1 (%), FEV1/FVC, DLCO (%), radiological stages, therapy for sarcoidosis and prednisone dosage to the presence of fragility fracture. All tests were two-sided, and *p* < 0.05 was considered statistically significant. All statistical tests were performed using SPSS 10.1 statistical software (SPSS 10.1).

## Results

**FIGURE 1:**
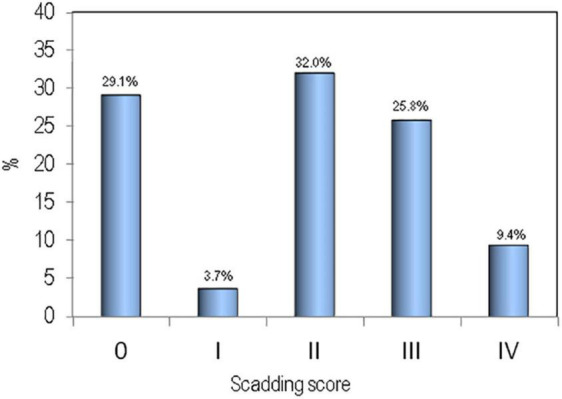
Distribution of the sarcoidosis population on the basis of radiological assessment by Scadding score.

The clinical and biochemical parameters of sarcoidosis patients and healthy controls are shown in Table 1. There were no significant differences between the two groups for age, BMI, calcium, phosphate, and vitamin D serum levels. Serum levels of creatinine and PTH were slightly higher in patients affected by sarcoidosis with respect to healthy controls (*p* < 0.05). Distribution of the sarcoidosis population on the basis of radiological assessment by Scadding score has been reported in Figure 1.

**TABLE 1:** Clinical and biochemical characteristics of the patients with sarcoidosis and in healthy controls.

	Sarcoidosis (*N* = 252)	Controls (*N* = 250)
Sex (F/M)	148/104	147/103
Age (years)	54.7 ± 12.1	56.6 ± 11.9
BMI (Kg/m^2^)	26.1 ± 4.5	26.0 ± 4.1
Creatinine (mg/dl)	1.12 ± 0.32	0.99 ± 0.12*
Calcium (mg/dl)	9.46 ± 0.86	9.26 ± 0.56
Phosphate (mg/dl)	3.53 ± 0.58	3.39 ± 0.63
Alkaline phosphatase (U/L)	71.25 ± 28.73	68.74 ± 21.33
25OHD (ng/ml)	23.44 ± 13.69	23.64 ± 12.9
PTH (pg/ml)	33.09 ± 11.9	26.40 ± 112.14*
FVC (%)	106.30 ± 19.94	—
FEV1 (%)	98.43 ± 19.89	—
DLCO (%)	84.10 ± 19.36	—
LS-BMD (g/cm^2^)	1.077 ± 0.160	1.102 ± 0.162
FN-BMD (g/cm^2^)	0.890 ± 0.140	0.888 ± 0.127
TH-BMD (g/cm^2^)	0.937 ± 0.146	0.950 ± 0.129

*p < 0.05 sarcoidosis vs. controls.

**FIGURE 2:**
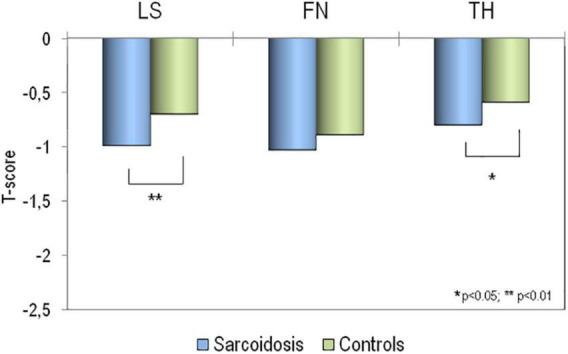
Values of BMD expressed as T-score at lumbar spine (LS), at femoral neck (FN) and at total hip (TH) in patients affected by sarcoidosis and in healthy controls. *p > 0.05; **p < 0.01 sarcoidosis vs. controls.

The mean values of BMD at all skeletal sites, in sarcoidosis patients and in healthy controls, expressed as T-score, have been reported in Figure 2. It is clear that BMD T-scores were lower in patients affected by sarcoidosis with respect to those obtained in healthy controls, but the difference was statistically significant only for BMD-LS (*p* < 0.01) and BMD-TH (*p* < 0.05).

Table 2 presents the age and BMI adjusted partial correlations of BMD values with lung function tests. In particular, BMD values at all skeletal sites were significantly associated with DLCO (%) (*p* < 0.05). Instead, we did not find any significant associations between BMD values with FVC (%) and FEV1 (%).

**TABLE 2:** Age and BMI adjusted partial correlations of BMD values and pulmonary tests in patients with sarcoidosis.

	*FVC (%)*	*FEV1 (%)*	*DLCO (%)*
*BMD-LS (g/cm^2^)*	0.05	0.07	0.20*
*BMD-FN (g/cm^2^)*	−0.04	−0.04	0.18*
*BMD-TH (g/cm^2^)*	−0.05	−0.02	0.17*

*p < 0.05.

Moreover, 77 patients with sarcoidosis and 43 healthy controls have reported a fracture. The most frequently reported sites for sarcoidosis patients were the vertebrae (*N* = 66), femur (*N* = 3), wrist (*N* = 1), ribs (*N* = 3), humerus (*N* = 1), and lower limbs (*N* = 3). In the healthy controls the previous fragility fracture site were the vertebrae (*N* = 9), wrist (*N* = 8), ribs (*N* = 2), pelvis (*N* = 1), and lower limbs (*N* = 23).

**FIGURE 3:**
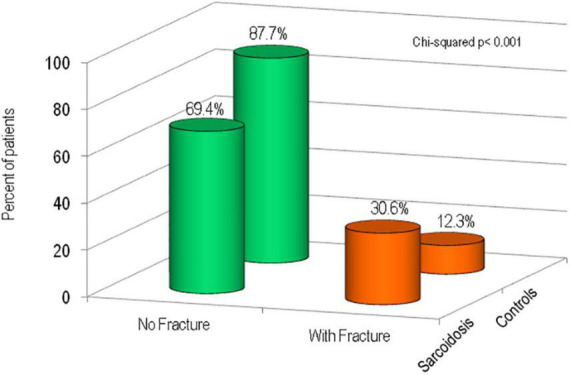
Percentage of sarcoidosis patients and healthy controls with fragility fractures.

**FIGURE 4:**
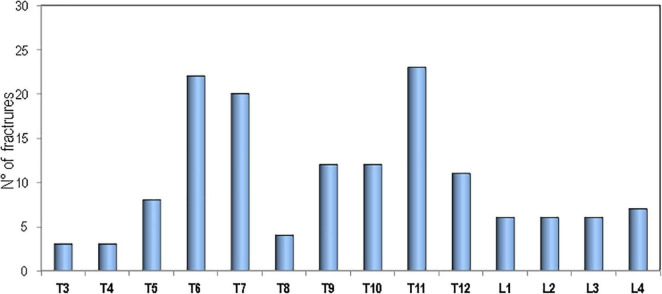
Distribution of vertebral fractures in sarcoidosis patients.

Figure 3 shows the percentage of patients affected with sarcoidosis and healthy controls on the basis of the presence of fragility fractures. It’s evident that the prevalence of fragility fractures was higher in patients with sarcoidosis than in healthy controls (30.6 vs. 12.3% respectively, *p* < 0.001). The prevalence of vertebral fractures in patients with sarcoidosis, by spinal location, is shown in Figure 4. The localization of vertebral fractures showed two peaks located at the level of the thoracic spine and in particular one at the level of T6-T7 and another at the level of T11.

Moreover, Table 3 shows the presence of osteoporosis/osteopenia and fragility fractures by grouping the sarcoidosis population on the basis of the ongoing pharmacological treatment. No significant differences are evident between the two groups.

**TABLE 3:** Presence of osteoporosis/osteopenia and fragility fracture grouped by sarcoidosis treatment.

Characteristics	Patients with sarcoidosis on treatment			
		Yes (*n* = 160)	No (*n* = 92)	*p*-value
Diagnosis osteoporosis	Osteoporosis	35/160 (21.7%)	14/92 (15.2%)	0.396
	Osteopenia	76/160 (47.8%)	45/92 (49.4%)	
	Normal	49/160 (30.4%)	33/92 (35.4%)	
Presence of fragility fracture		51/160 (31.9%)	29/92 (31.6%)	0.953

Dichotomous variable, reference category no (Chi square yates corrected test).

**FIGURE 5:**
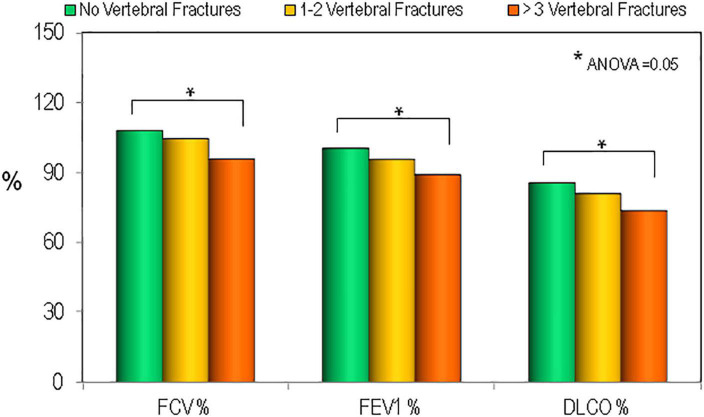
Values of pulmonary tests in patients affected by sarcoidosis by the presence and the severity of vertebral fractures. *p < 0.05 between groups.

In order to define the relationship between the respiratory parameters and vertebral fracture burden we evaluated the average lung functionally measurements in the sarcoidosis population divided according to the numerous of vertebral fractures. As reported in Figure 5 the patients with ≥3 vertebral fracture had lower values of FVC (%), FEV1 (%), and DLCO (%) (ANOVA = 0.05). In multiple regression analysis, statistically significant associations were found for predictors of vertebral fractures. In particular, multiple regression analyses showed that BMI was positively associated with fragility fracture, while BMD-TH, DLCO(%) and therapy use was negatively associated (Table 4).

**TABLE 4:** Multiple linear regression analysis of predictors for the vertebral fracture in patients affected of sarcoidosis.

Variable	Unstandardized coefficient, b	95% CI	*p*
**Vertebral fracture**			
BMD-TH	–1.038	−1.717 to −0.359	0.003
BMI	0.021	0.002 to 0.039	0.029
DLCO%	–0.006	−0.011 to −001	0.013
Therapy for sarcoidosis	–0.191	−0.377 to −0.005	0.044

Whole set of variables included into the model: sex, age, BMI, creatinine, vitamin D, BMD-LS, BMD-FN, BMD-TH, FVC (%), FEV1 (%), FEV1/FVC, DLCO (%), Radiological stages, Therapy for sarcoidosis, prednisone dosage.

## Discussion

To our knowledge this is the first single-center study that evaluated the presence of fragility fractures in sarcoidosis patients compared to matched controls.

This study shows that fragility fractures are significantly more frequent in patients with sarcoidosis than in control subjects (30.6 vs. 12.3%). Similarly, a previous French study of 142 sarcoidosis patients reported that fragility fractures occurred in 23.5% of patients (17). In our study we observed a marked increase in vertebral fractures, while the prevalence of non-vertebral fractures was not different compared to the control group.

Similarly, in the Maastricht study at baseline 20% of patients with sarcoidosis had vertebral fractures while non-vertebral fractures were rare (12). A subsequent 4-year follow-up study reported that the prevalence of vertebral fractures increased from 20 to 32%, while non-vertebral fractures remained infrequent (16). Moreover, the study by Bours et al., carried out using a database of general practitioners across the United Kingdom, reported that the risk of any fractures was similar in patients with sarcoidosis and in matched controls while the risk of vertebral fractures was significantly increased and the risk of non-vertebral fractures marginally reduced (20). In our study population, vertebral fractures in patients with sarcoidosis predominantly affect the thoracic spine (with peaks at the level of T6, T7, and T11) and therefore, with a distribution similar to that which we have previously encountered in patients with severe pulmonary interstitial diseases and in waiting lung transplant (28). The prevalent distribution of thoracic fractures may negatively influence the course of the disease because these fractures, especially those of moderate/severe degree, have deleterious effects on pulmonary functionally tests by reducing lung volume contributing to a restrictive ventilatory defect (29–31). In our study population, patients with sarcoidosis who have a higher number of vertebral fractures have also a reduction in pulmonary tests values and in particular in DLCO and FVC. A similar link between vertebral fracture loading and pulmonary tests was observed in a previous study in patients with severe pulmonary fibrosis (32). The fact that the increase in fragility fractures in our study is due to vertebral fractures while non-vertebral fractures do not differ from those of control subjects indicates that sarcoidosis may have a negative impact mainly on skeletal site where trabecular bone prevails.

At present, the role of the possible pathophysiological mechanisms responsible for increased bone fragility in sarcoidosis has not yet been clarified. The reduction of BMD values is generally considered one of the fundamental determinants of bone fragility and fractures. Instead, most of the literature data reported that patients with sarcoidosis had normal BMD values. Furthermore, the Maastricht longitudinal study showed that the increase in vertebral fractures during the 4 years of follow-up was not associated with changes in BMD (16) and a small New Zealand study found no changes in BMD over 1–2 years, regardless of baseline vitamin D levels (18).

In our study, the small but significant reduction in lumbar and femoral BMD values in patients with sarcoidosis compared to healthy controls appears to be in agreement with some previous studies (14, 15) but in contrast with most of the others (16–18). This finding can be explained by the fact that our center, being a referral center for sarcoidosis, manages patients in more advanced stages of the disease and, therefore, are more frequently characterized by a significant lung volume or lung diffusion capacity impairment. This hypothesis appears to be supported by the correlation we observed between BMD values and pulmonary tests particularly DLCO. An important aspect of our study is the assessment of risk factors associated with the presence of vertebral fragility fractures. In fact, the multiple regression analysis showed that in addition to BMD-TH and DLCO also treatment therapy for sarcoidosis were significantly associated with vertebral fractures. The key therapy of sarcoidosis is represented by glucocorticoid and it is well known how chronic use of these drugs can lead to secondary osteoporosis (33). Indeed, the role of glucocorticoid treatment on bone fragility in patients with sarcoidosis has not yet been elucidated. Some studies found an increased risk of any fracture among sarcoidosis patients treated with GCs compared with non-users (20, 21) but in other studies BMD was normal also in patients treated with glucocorticoids (12, 17). Moreover, the recent study by Bours et al reported that in sarcoidosis patients, both the higher dose and the cumulative dose of glucocorticoids did not significantly increase the risk of fracture (20). The heterogeneity of the data related to the treatment with glucocorticoids can be explained by the fact that the glucocorticoids, in inflammatory diseases such as sarcoidosis, reduce the cytokine release and the inflammatory status which can partially compensate their direct negative effect on bone (20, 34).

However, glucocorticoid therapy may represent an element of risk for fracture even independently of BMD. In fact, it is known that glucocorticoids have a negative effect on bone quality as they reduce new bone formation (21). Thus, our data supports the intriguing hypothesis that sarcoidosis activity itself may represent a major risk factor for vertebral fractures and underlines how crucial is a early diagnosis and severity assessment of disease to promptly achieve a proper treatment, even with glucocorticoids. In this study, multiple regression analysis showed that BMI is the only parameter with a significant protective effect against vertebral fractures in patients with sarcoidosis. This finding seems to be in agreement with a previous study that reported a strong association between reduction in BMI and the risk of vertebral fractures in COPD patients (35). Moreover, Cremers et al. reported that sarcopenia is frequent among sarcoidosis patients and could increase the risk of falls and fragility fracture (36).

This study presents some limitations. Firstly, the impossibility of establishing any causal relationship between the parameters in consideration of the cross-sectional design of the study. Secondly, no information on muscle mass or strength were collected. Thirdly, the lack of data about markers of bone turnover and inflammation. Nevertheless, our study presents several strengths. Firstly, the fact that a large sample size of sarcoidosis patients were evaluated and compared with healthy controls. Secondly, to our knowledge, this is the first study that evaluated factor associated with vertebral fracture in sarcoidosis subjects.

## Conclusion

Our data show that vertebral fractures represent a frequent complication in patients with sarcoidosis. The degree of severity of the sarcoidosis disease appears to be the main determinant of bone fragility. Therefore, an assessment of bone status should be recommended in patients with sarcoidosis in order to define the fracture risk and initiate adequate therapy to prevent vertebral fractures.

## Data availability statement

The raw data supporting the conclusions of this article will be made available by the authors, without undue reservation.

## Ethics statement

The studies involving human participants were reviewed and approved by the Institutional Review Board of Siena University Hospital. The patients/participants provided their written informed consent to participate in this study.

## Author contributions

SG and AA: conception and design. CC, EG, GM, CM, CO, and YN: acquisition of data. SG, CC, and PC: drafting the manuscript. SG and CC: statistical analysis. CC, PC, and SG: analysis and interpretation of data. EB and SG: critical revision. All authors contributed to the article and approved the submitted version.
